# Supplementary material for: FapR regulates HssRS-mediated heme homeostasis in Bacillus anthracis
Source: mBio. 2025 May 23;16(6):e02057-24. doi: 10.1128/mbio.02057-24 (PMC12153329; doi:10.1128/mbio.02057-24)
Supplement: Supplemental material — Supplemental tables and figures. [file mbio.02057-24-s0001.docx]

**FapR regulates HssRS-mediated heme homeostasis in *Bacillus anthracis***

Hualiang Pi^1,2,3^, Owen S. Burroughs^1,2^, Sophia M. Carlin^1,2^, William N. Beavers^1,2^, Gideon H. Hillebrand^4^, Evan S. Krystofiak^5^, Devin L. Stauff^4^, and Eric P. Skaar^1,2, #^

^1^Vanderbilt Institute for Infection, Immunology, and Inflammation, Vanderbilt University, Nashville, TN

^2^Department of Pathology, Microbiology, & Immunology, Vanderbilt University Medical Center, Nashville, TN

^3^Current address: Department of Microbial Pathogenesis and Microbial Sciences Institute, Yale University School of Medicine, New Haven, CT

^4^Department of Biology, Grove City College, Grove City, PA

^5^Department of Cell and Developmental Biology, Vanderbilt University, Nashville, TN

^#^Address correspondence to [eric.skaar@vumc.org](mailto:eric.skaar@vumc.org)

**SUPPLEMENTAL INFORMATION**

**Table S1. Isolated spontaneous resistant suppressors with frameshift mutations in *fapR*.**

| **Background** | **Suppressor** | **Allele** | **Genotype** |
| --- | --- | --- | --- |
| WT  *bas3009*::P*_hrt_-relE* *bas4599*::P*_hrt_-relE* | S1, S2, S4 | *fapR-1* | Deletion of A at nucleotide 10  🡪 frameshift after amino-acid residue 3 |
|  | S3 | *fapR-2* | Deletion of T at nucleotide 279  🡪 frameshift after amino-acid residue 92 |
|  | S5, S6 | *fapR-3* | Deletion of C at nucleotide 322  🡪 frameshift after amino-acid residue 107 |

**Table S2. Strains and plasmids used in this study**

| **Species** | **Genotype** | **Description** | **Antibiotic resistance** | **Reference** |
| --- | --- | --- | --- | --- |
| *B.*  *anthracis*  strain  Sterne | WT | Wildtype laboratory stock | none | Lab stock |
|  | 2xRelE (*bas3009*::P*_hrt_-relE* *bas4599*::P*_hrt_-relE*) | RelE selection strain (*hrt* promoter fused to relE and inserted into the BAS3009 and BAS4599 pseudogene loci | none | (38) |
|  | *fapR*::*tet* | The *fapR* reading frame replaced with a tetracycline cassette | Tet | This study |
|  | *fapR*::*tet* pOS1.P*_lgt_fapR* | Complementation *fapR* deletion *in trans* | Tet, Cam | This study |
|  | Δ*hemX* | Lacking the *hemX* reading frame | none | This study |
|  | Δ*hemX* pOS1.P*_lgt_hemX* | Complementation Δ*hemX* deletion *in trans* | Cam | This study |
|  | *fapR*::*tet* Δ*hemX* | Double mutant of *fapR* and *hemX* | Tet | This study |
|  | WT pOS1.P_lgt_*hssS*.*GFP* | WT harboring a GFP-HssS protein fusion construct. | Cam | This study |
|  | *fapR* pOS1.P_lgt_*hssS*.*GFP* | *fapR*::*tet* harboring a GFP-HssS protein fusion construct. | Tet, Cam | This study |
|  | WT pOS1 P*_hrt_xylE* | WT harboring a XylE reporter | Cam | This study |
|  | *fapR*  pOS1 P*_hrt_xylE* | *fapR*::*tet*  harboring a XylE reporter | Tet, Cam | This study |
|  | *hemX* pOS1.P*_hrt_xylE* | Δ*hemX* harboring a XylE reporter | Cam | This study |
|  | *fapR hemX* pOS1.P*_hrt_xylE* | *fapR*::*tet* Δ*hemX* harboring a XylE reporter | Tet, Cam | This study |
|  | WT  pOS1 P*_hrt_*mcherry | WT harboring an mcherry reporter | Cam | This study |
|  | *fapR*  pOS1 P*_hrt_*mcherry | *fapR*::*tet*  harboring an mCherry reporter | Tet, Cam | This study |
|  | *hemX* pOS1.P*_hrt_*mcherry | Δ*hemX* harboring an mCherry reporter | Cam | This study |
|  | *fapR hemX*  pOS1.P*_hrt_* mcherry | *fapR*::*tet* Δ*hemX* harboring an mCherry reporter | Cam | This study |
| *E.*  *coli* | DH5α WT | Wildtype laboratory stock for cloning | Tet, Cam | Lab stock |
|  | K1077 WT | Wildtype laboratory stock for cloning | none | Lab stock |
| **Plasmid** | **Description** | |  | **Reference** |
| pLM4 | Plasmid for gene deletion | | Kan | Lab stock |
| pOS1 | Plasmid for complementation | | Cam | Lab stock |

| **Primer name** | **Sequence** | **Use** |
| --- | --- | --- |
| fapR_fwd | aaatacaattgaggtgaacaATGAAAAAAAGAAGAAGTAAAAAAG | Gene complemen-tation |
| fapR_rev | caccaccaccTAAGTTGTTACCTTCCTCAC |  |
| 3FLAG_hemX_F | ttggtatttaGGTGGTGGTGGTGGTGATTATAAG |  |
| hemX_fwd | aaatacaattgaggtgaacaATGAGTTTTTTAAATAACAGTATTATTTATC |  |
| hemX_rev | caccaccaccTAAGTTGTTACCTTCCTCAC |  |
| 3FLAG_fapR_F | taacaacttaGGTGGTGGTGGTGGTGATTATAAG |  |
| fapR_ck_fwd | ctcgattaaagggaggcgacaa |  |
| fapR_ck_rev | cagcctttggagcatgatcgc |  |
| fapR_fwd2 | aaatacaattgaggtgaacatATGAAAAAAAGAAGAAGTAAAAAAG |  |
| fapR_rev2 | aaacactacccccttgtttggatccTCATAAGTTGTTACCTTCC |  |
| hemX_fwd | aaatacaattgaggtgaacatATGAGTTTTTTAAATAACAGTATTATTTATC |  |
| hemX_rev | aaacactacccccttgtttggatccTTATAAATACCAAAAGTGGAAGTTG |  |
| fapR_5_XmaI | CCATGACCCGGGATTACTGCATTTGGAGAGATGG | Gene deletion |
| fapR_P2_StuI_SOE | GATTTTCATAGGCCTACCGCACCATCCATTTTTATGAC |  |
| fapR_P3_StuI_SOE | GTGCGGTAGGCCTATGAAAATCGCAATAGATGCAATG |  |
| fapR_3_SacI | GCATGAGAGCTCGGAGCTTTCAATCCAAATAATGCC |  |
| hemX_5_XmaI | CCATGACCCGGGTTATGCTGCTGTTGAGTTAGG |  |
| hemX_P2_StuI_SOE | CTCTCCTCAAGGCCTCCGATCATCCTTCCAAATTATAAAG |  |
| hemX_P3_StuI_SOE | GATCGGAGGCCTTGAGGAGAGAAAATTATGCGC |  |
| hemX_3_SacI | GCATGAGAGCTCGATCAGTTCCTACTACTGTCTC |  |
| fapR_CHK1 | TCCAGACGTATTATTTATGACAGC | Deletion verification |
| fapR_CHK2 | AATATCGATTGATCATTAGAAGAACC |  |
| hemX_CHK1 | CGCACTCAGAAACGACAATTGG |  |
| hemX_CHK2 | CGGTCCGCAGCCTCTAATCC |  |
| Peds_fwd | gccttaaagacgatccggggTATCTTACCCGATTGTATCATGTTTATTTTCGTC | pOS1.Peds Reporter |
| Peds_rev | tagaaaccatCTCTCCACCCCCGCTGTT |  |
| mCherry_fwd | gggtggagagATGGTTTCTAAAGGAGAAG |  |
| mCherry_rev | aaacactacccccttgtttgTTATTTATATAATTCATCCATTCCG |  |

**Table S3. Oligos used in this study**


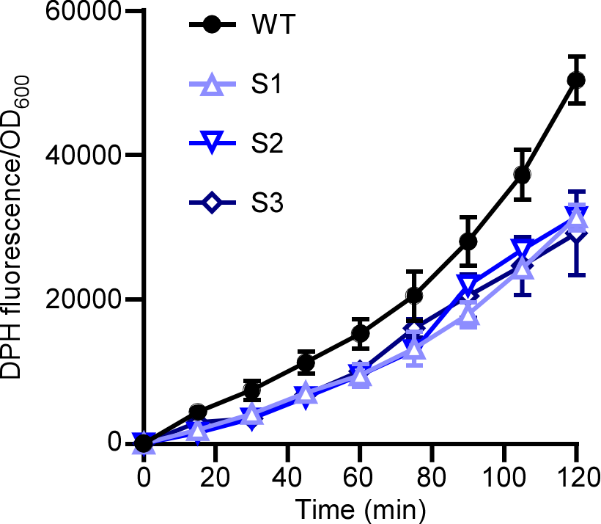


**Figure S1. Disruption of *fapR* leads to increased membrane rigidity in *B. anthracis*.** Membrane permeability was evaluated over 2 h in *B. anthracis* Sterne WT and the three representative suppressors by monitoring the fluorescence of 1,6-diphenyl-1,3,5-hexatriene (DPH) (excitation, 365 nm; emission, 460 nm). All data represent means ± SD for measurements acquired in three biological replicates. Statistical analysis was performed by comparing each isolate to the WT strain using area under the curve followed by a t-test. All p-values were less than 0.05.


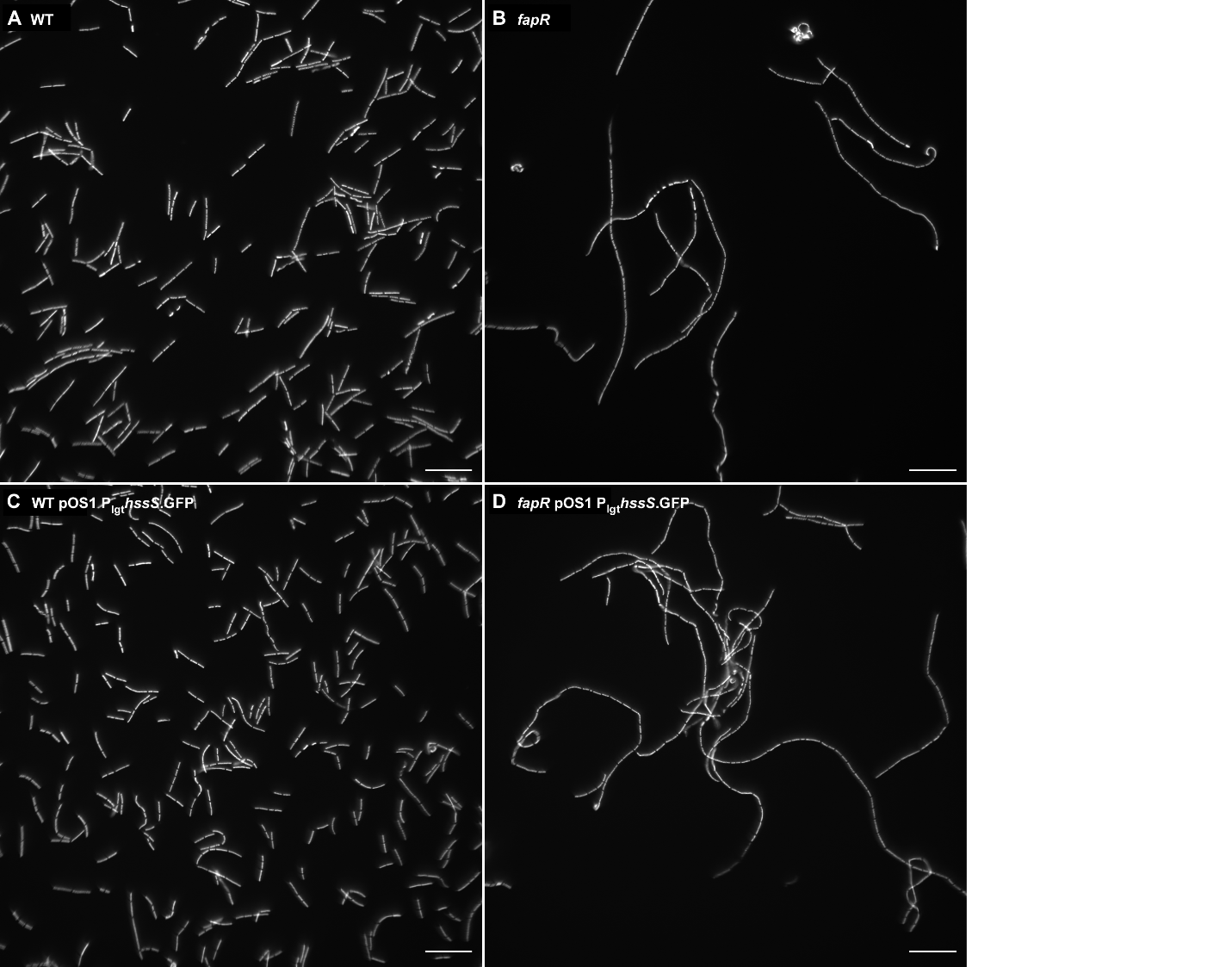


**Figure S2**. **Deletion of *fapR* leads to formation of long curved filaments.** Representative fluorescent images of WT, *fapR*, WT pOS1.P_lgt_*hssS*.*GFP*, and *fapR* pOS1.P_lgt_*hssS*.*GFP*. Cells were grown to OD_600_~1 prior to staining with Hoechst. Scale bar, 20 µm.


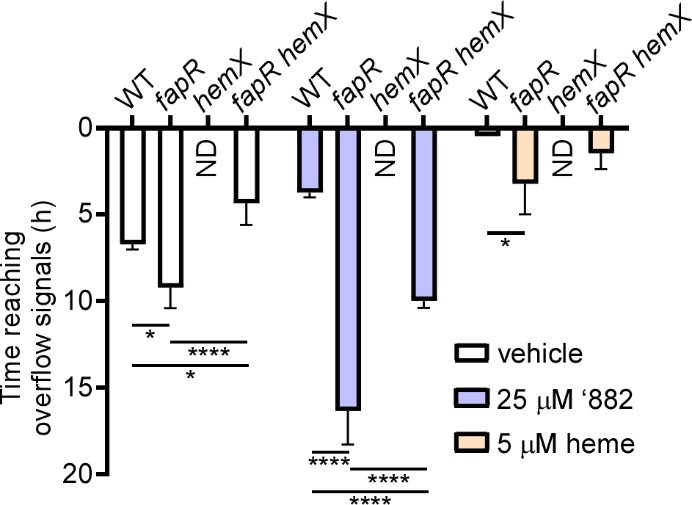


**Figure S3. Heme sensing response is highly activated in Δ*hemX* but compromised by deletion of *fapR*.** Overnight cultures of *B. anthracis* strains harboring pOS1. P*_hrt_*-*mcherry* were inoculated in LB amended with varied compound as indicated and fluorescence was monitored for 20 h at 37 ^o^C (Ex: 590 nm; Em: 620 nm). The time taken to reach an overflow signal, indicating maximum fluorescence beyond the detection limit, serves as a proxy for P*_hrt_* promoter activation regulated by the HssRS system. The fluorescence signal in the *hemX* mutant reached its maximum instantly so the time required to reach an overflow signal in this mutant is not determined (ND). All data are mean ± SEM (n=9). Statistical analyses were done using two-way ANOVA ∗P < 0.05, ∗∗P < 0.01, ∗∗∗P < 0.001, and ∗∗∗∗P < 0.0001.


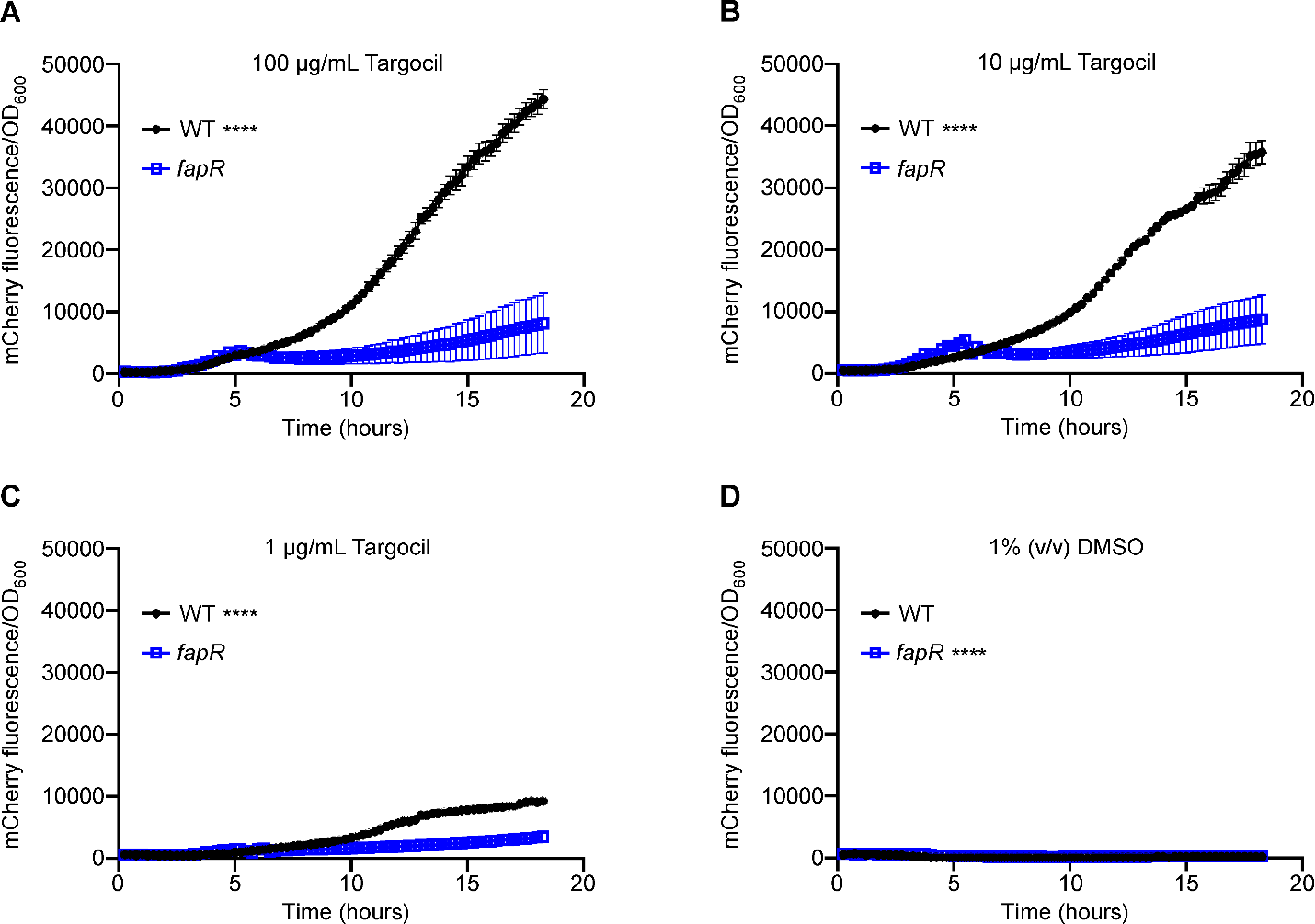


**Figure S4: Disruption of *fapR* results in impaired activation of the EdsRS two-component system.** *B. anthracis* strains harboring pOS1.P*_eds_*-mCherry, a fluorescent reporter of the EdsRS two-component system*,* was grown at 37 °C in (**A)** 100 µg/mL, (**B**) 10 µg/mL, or (**C**) 1 µg/mL of the known EdsRS activator targocil or in (**D**) 1% (v/v) DMSO. Fluorescence of mCherry (ex. 579, em. 616) and OD_600_ were measured at 15-minute intervals for 18 hours. All data represent means ± SEM for measurements acquired in three biological replicates. Statistical analysis was performed using area under the curve (AUC) followed by an unpaired t-test ∗P < 0.05, ∗∗P < 0.01, ∗∗∗P < 0.001, and ∗∗∗∗P < 0.0001. Significance symbols follow the genotype with the greater AUC.
